# State machine design for an automated peritoneal dialysis machine

**DOI:** 10.3389/fmedt.2025.1630829

**Published:** 2025-08-29

**Authors:** Wafa A. Baroudi, Fatimah B. Alnahdi, Raghad S. Aljohani, Maryam A. Alzuabi, Nora K. Alsaqoub, Ibrahim A. Aljamaan, Naif A. Alrubai, Sajid Ali

**Affiliations:** ^1^ Biomedical Engineering Department, College of Engineering, Imam Abdulrahman Bin Faisal University, Dammam, Saudi Arabia; ^2^ Department of Mechanical and Energy Engineering, College of Engineering, Imam Abdulrahman Bin Faisal University, Dammam, Saudi Arabia

**Keywords:** state machine design, automated peritoneal dialysis (APD), peristaltic pump, biomechanics, hemodialysis, chronic kidney disease (CKD), dialysis solutions

## Abstract

According to the National Library of Medicine (NLM), chronic kidney disease (CKD) affects more than 10% of the world's population, and peritoneal dialysis (PD) is one of the promising treatments. Despite the advantages of the current PD machine over alternative treatments, it has certain limitations, such as high consumable costs, lengthy daily sessions, and a lack of portability. This work aims to implement a finite state machine design to modify the process of an automated, economical PD system. The proposed optimized process includes a flush system stage, where tubes are rinsed before and after each session to avoid contamination. This design is intended for use by only one patient to prevent contamination. Furthermore, a turbidity sensor is added to measure the efficiency of the dialysis process and reduce the current dialysis time, which can reach eight hours. The finite state machine design is developed using LabVIEW software, featuring a user-friendly interface that allows users to track the process's progress.

## 1 Introduction

In 2015, the Saudi Ministry of Health (MOH) reported that the number of individuals suffering from kidney failure in Saudi Arabia exceeded 15,000, with over 850 million affected worldwide (1–3). Kidney failure patients lose 85%–90% of their kidney function, which significantly impairs the kidneys’ ability to maintain the body's internal balance, filter blood, and regulate blood pressure (4–6).

Various treatment methods for kidney failure include peritoneal dialysis (PD), hemodialysis (HD), and kidney transplantation (7, 8). PD is a treatment option that utilizes the abdominal cavity (peritoneum) as a natural filter. This is achieved by filling the abdomen with a dialysis solution through a catheter, which drains waste products and excess fluids. Unlike other treatments, PD can be performed at home, eliminating the need for dialysis centers or hospitals (9, 10). Despite its advantages over alternative treatments, the current PD machine has limitations, including high costs due to disposable tubes, a rigid interface, a lack of portability, and lengthy daily sessions (11–15).

This work aims to design a finite state machine for an optimized PD system that modifies the process with a fully monitored, automated, user-friendly interface. This design significantly minimizes costs by utilizing reusable tubes, assuming that only one user per machine is involved, and reduces session duration by employing a turbidity sensor to terminate the process as soon as the drainage is clean. Additionally, the proposed interface offers three levels of accessibility based on user needs, whether the user is a patient, doctor, or developer.

## 2 Physiology

### 2.1 The kidney’s function

**Figure 1:**
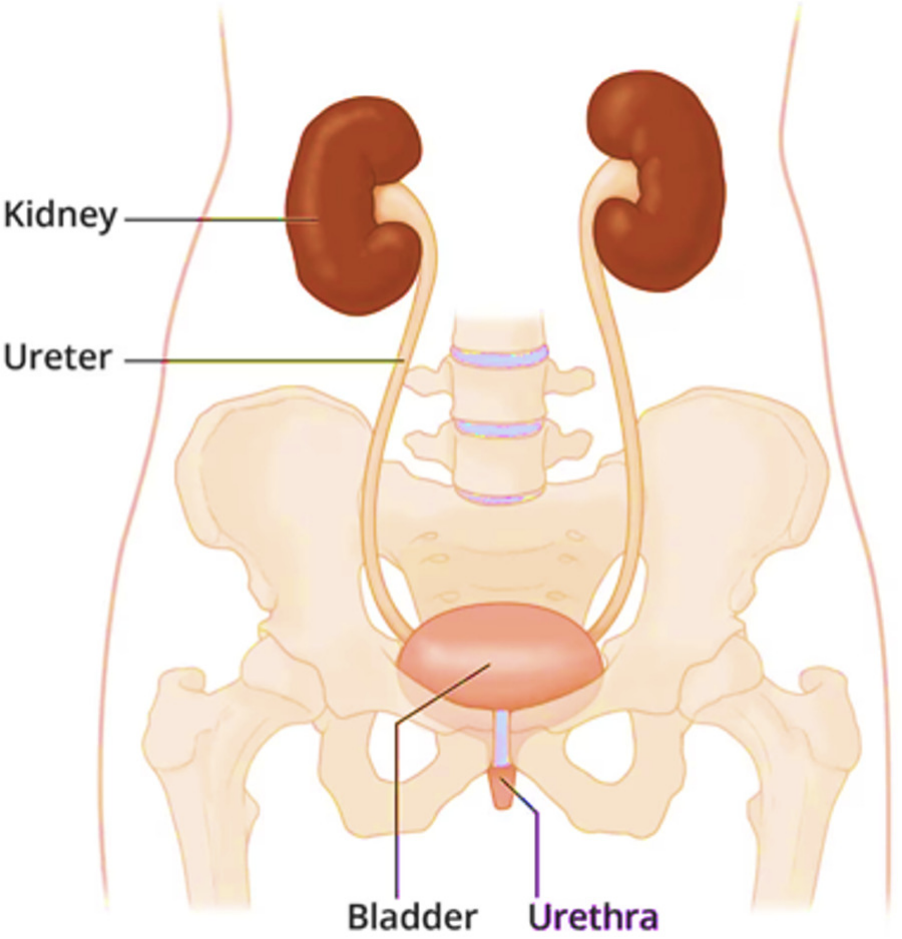
The location of the kidneys (40).

The kidneys are a pair of bean-shaped organs, each approximately the size of a fist, located on either side of the spine, just below the rib cage, as shown in Figure 1. According to the National Institute of Diabetes and Digestive and Kidney Diseases (NIDDK), around one in every 2,000 individuals is born each year with a single kidney worldwide (16–18). In cases where a single kidney functions well, individuals may not even be aware that they have one kidney (19).

The primary role of the kidneys is to maintain the body's internal balance (homeostasis). They serve as a filtration system, continuously filtering approximately half a cup of blood per minute to excrete excessive water and waste products as urine. The most common waste products include sodium, potassium, bicarbonate, calcium, and phosphate (20, 21). In addition to their filtration function, the kidneys also perform essential roles such as releasing hormones to regulate blood pressure, stimulating red blood cell production in the bone marrow, and maintaining a healthy skeletal system (22).

### 2.2 Kidney diseases

Kidney diseases can lead to renal failure, a condition in which the kidneys fail to perform their functions. There are two types of kidney disease: Acute Kidney Injury (AKI) and CKD (23).

AKI occurs rapidly, over hours or days, when the kidneys suddenly lose their ability to remove waste from the blood and produce urine. This condition can develop quickly due to factors such as extreme dehydration, severe infections, and medications toxic to the kidneys. With appropriate treatment, kidney function is usually restored within days or weeks, and it may not require kidney replacement therapy (transplant or dialysis). However, AKI can sometimes lead to CKD (24, 25).

CKD, on the other hand, progresses slowly over the years. The damage to the kidneys cannot be reversed but it can be delayed, and kidney function declines until the kidneys no longer function at all. This condition is then called end-stage renal disease (ESRD). CKD can be caused by several factors, the main ones being Diabetes, nephritis (inflammation of kidney tissue), glomerulonephritis (inflammation of the filter t tissue in the kidney), hypertension (medical term for high blood pressure), and atherosclerosis. When a patient reaches ESRD, available treatment options include kidney transplants, HD, and PD (24, 25).

### 2.3 Hemodialysis vs. peritoneal dialysis

The two main types of dialysis are PD and HD, each with distinct properties and procedures. Hemodialysis is a treatment method where blood is filtered outside the patient's body using a hemodialysis machine, which functions like an artificial kidney with a special filter, often referred to as a dialyzer. The hemodialysis method involves a filter that separates blood into two sections via a permeable membrane (26–28). Table 1 presents a comparison between HD and PD across various aspects.

**Table 1:** Brief comparison between hemodialysis and peritoneal dialysis.

Dialysis types	Hemodialysis	Peritoneal dialysis
Blood filtration process	Conducted outside the body	Conducted inside the body
Used filter	Special filter manufactured with specific specifications	Natural filter, which is the peritoneum membrane
Location	Hospitals or clinical centers	Can be performed at home, hospitals, or clinical centers
Duration	Three times a week, four hours per session	Daily, eight to 12 h per exchange
Pre-dialysis surgery	An arteriovenous fistula surgery is needed	Implanting catheter surgery is needed

### 2.4 The evolution of peritoneal dialysis

**Figure 2:**
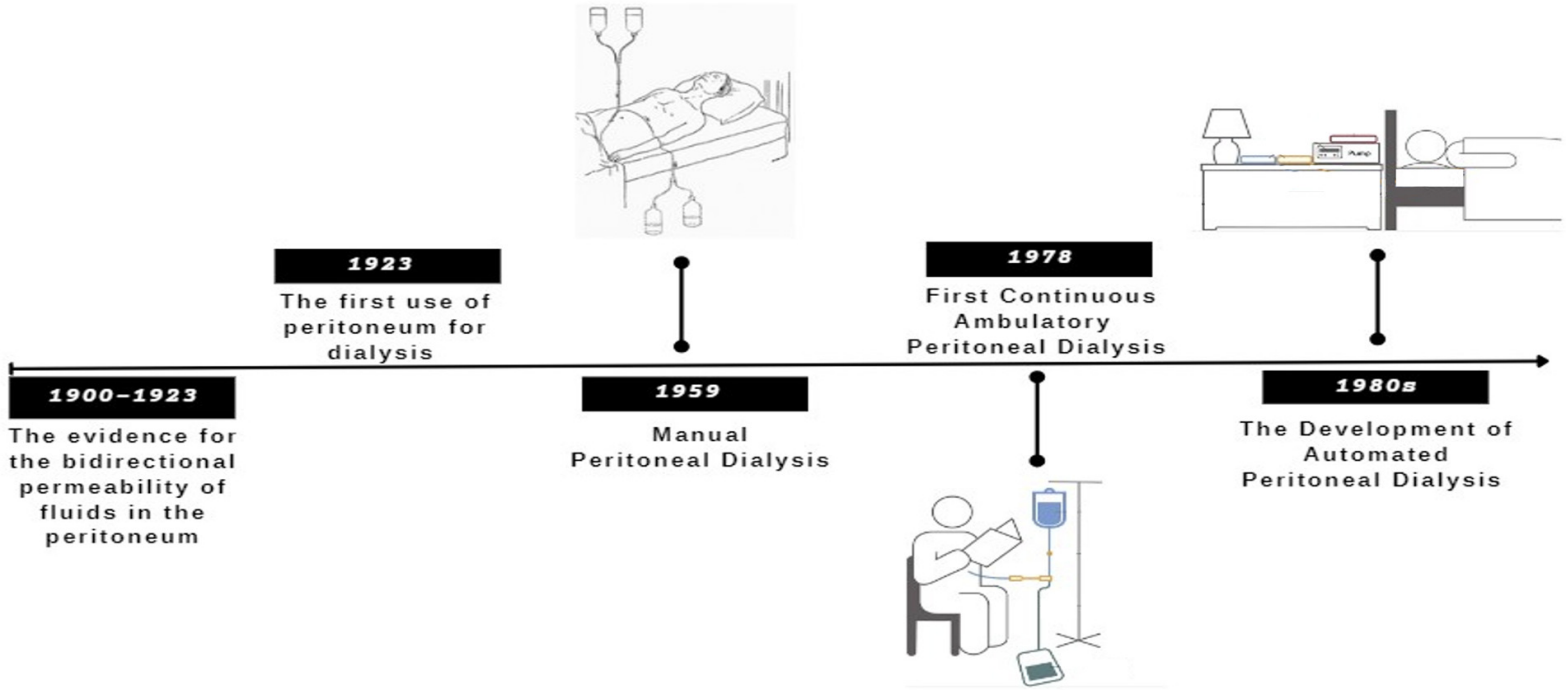
Summary of the important PD evolution timeline.

The timeline for the evolution of peritoneal dialysis is illustrated in Figure 2 (29–32).

## 3 Proposed design

### 3.1 Concept design

**Figure 3:**
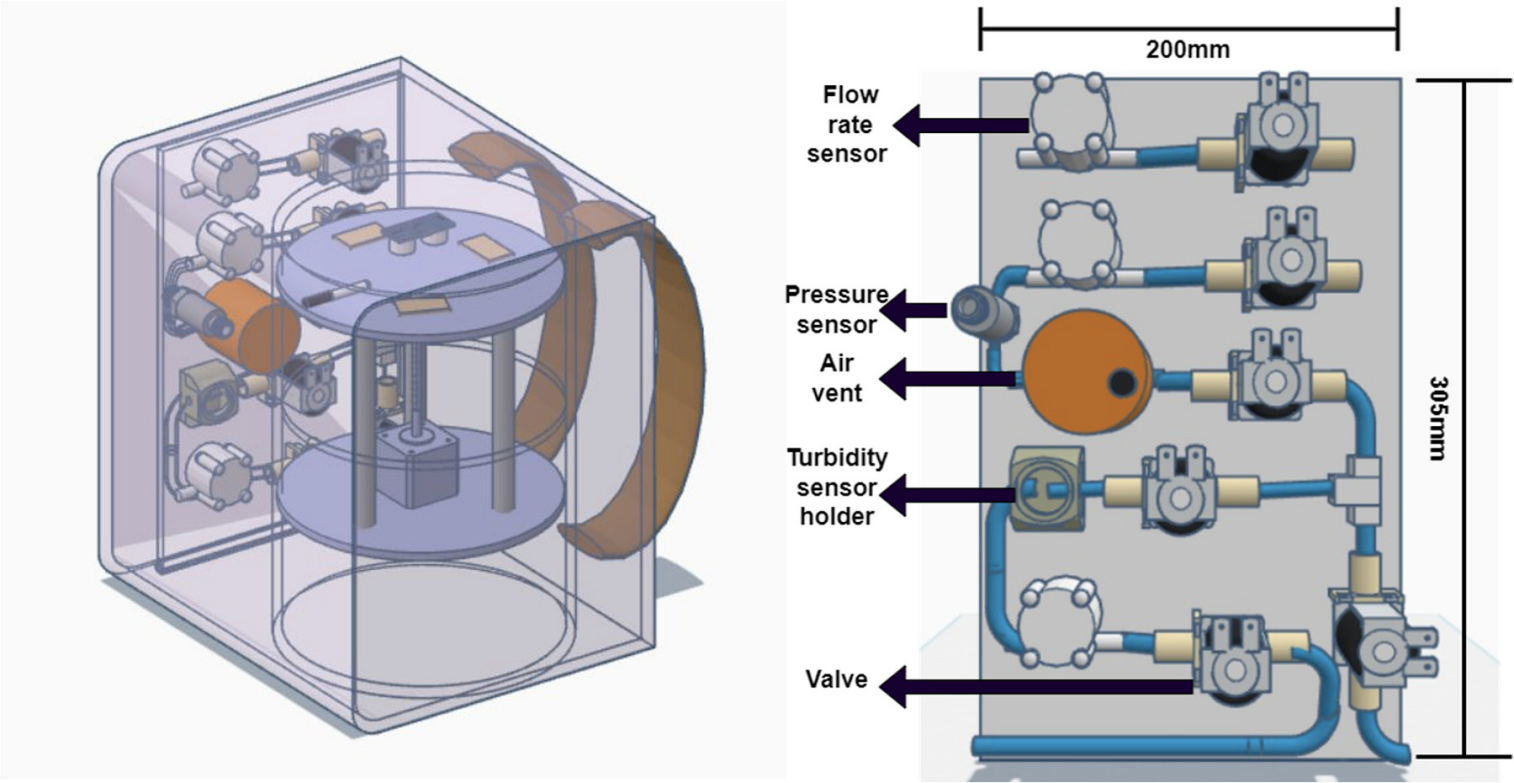
The proposed design on the left and a simple sketch of how the electrical components will be organized on a board.

**Figure 4:**
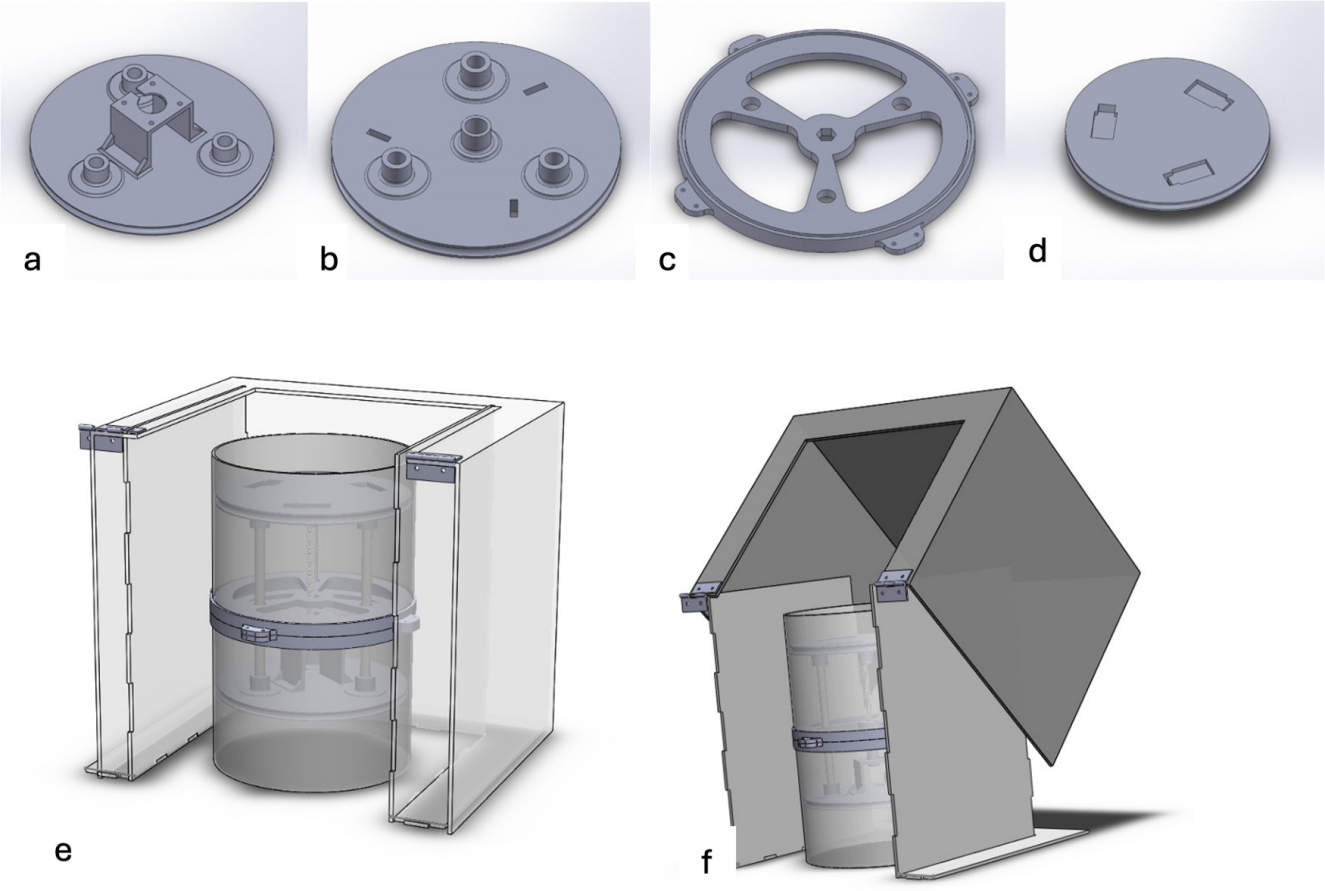
**(a)** Lower Plate in final design, **(b)** upper plate in final design, **(c)** the middle plate, **(d)** upper plate with heater cavities, **(e)** the design of cover, **(f)** the movement of cover.

**Figure 5:**
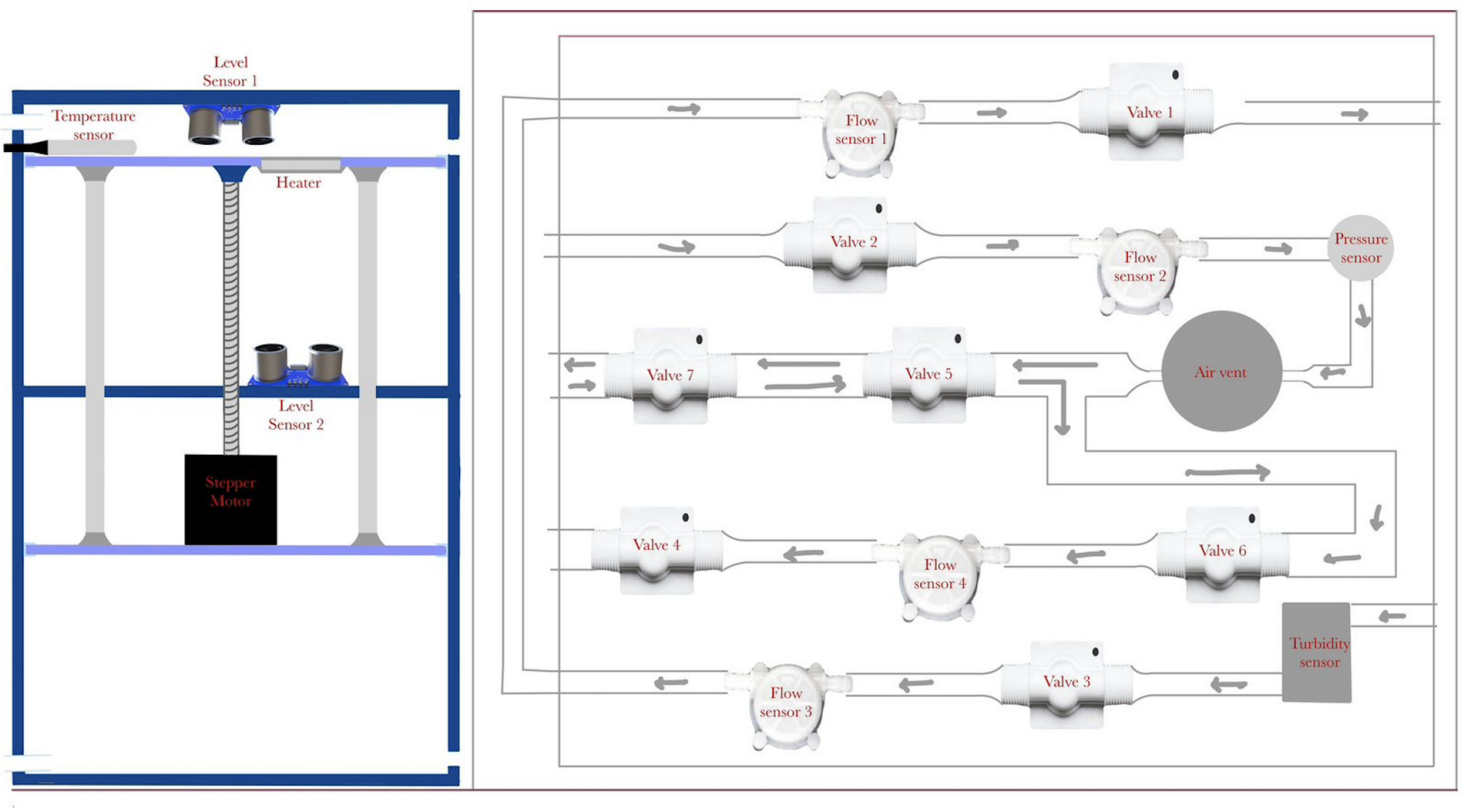
Simple sketch for the first box design combined with the tank part.

**Figure 6:**
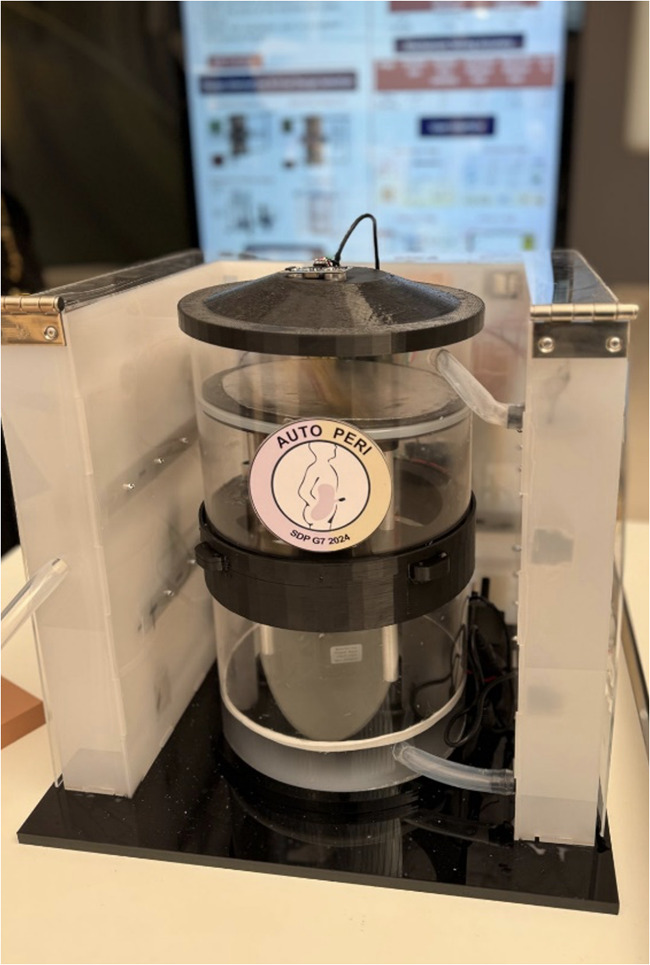
The final prototype as per the proposed design.

The state machine design is applied to an optimized automated peritoneal dialysis machine and process. This proposed system consists of two main components: the physical PD prototype and a simulation user interface. The physical design is illustrated in Figures 3, 4. Firstly, the design contains an electrical components board where all the sensors, tubes and circuit are organized to fit safely on the board. Secondly, a backpack with a belt to cover the catheter insertion place to avoid infection from surrounded environment are included. Thirdly, two tanks with 1l of volume for each are used with applying the newly designed mechanism to achieve the dialysis process. The tanks and electrical board will be placed inside the portable backpack that the patient can carry anywhere. The design of the new mechanism employed in both tanks is shown in Figures 5, 6.

Around the two-tank prototype, there is a cover that contains the pipes (solution path) with solenoid valves to control the flow of the solution. Several sensors are incorporated to measure different parameters, including flow meters, a pressure sensor, a level sensor, a turbidity sensor, and an air vent.

**Figure 7:**
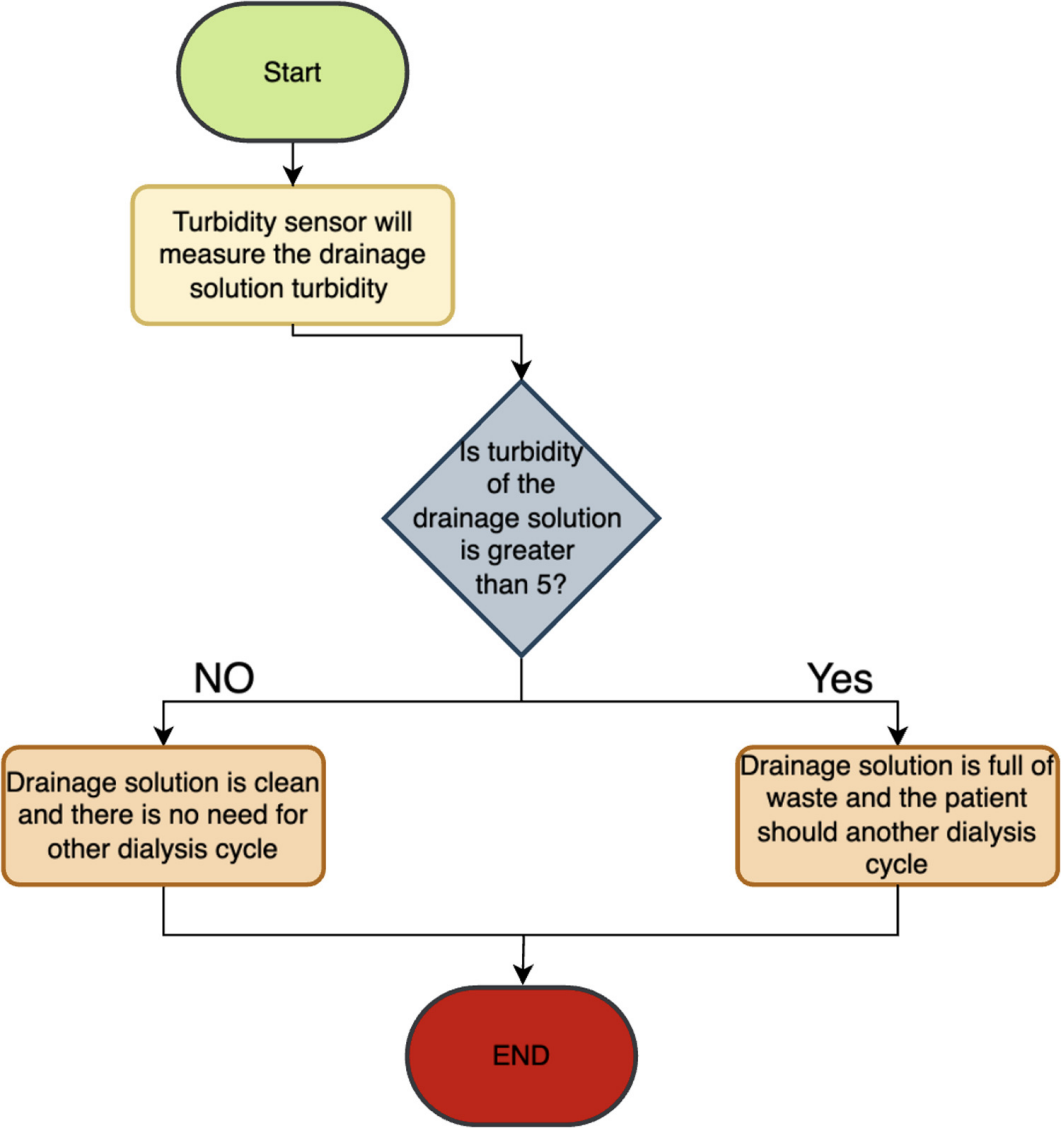
Flowchart showing how the turbidity sensor work.

The turbidity sensor measures the efficiency of the dialysis procedure by assessing the waste turbidity after each batch, helping to determine the need to inject more solution and continuing the dialysis session or stopping the session if the dwelled solution is clear. This will help in reducing session time. Figure 7 shows a flowchart that illustrate how the function of the turbidity.

While turbidity provides a useful approximation of waste concentration, it may not fully reflect the clearance of large molecular weight toxins such as β2-microglobulin. As a result, the current system design incorporates a confirmation step to avoid premature termination, where turbidity thresholds must persist over a defined time window. This strategy minimizes the risk of incomplete dialysis. For future versions, modeling of uremic toxin kinetics, especially for macromolecules, will be integrated to ensure clearance targets are met, even during shortened treatment durations.

The simulation interface allows for monitoring fluid movement, sensor readings, and sending alarms to halt the process in case of any errors. It is designed to create a fully monitored and controlled environment to minimize device errors and implement safety precautions. The flush system was specifically calibrated to meet sterilization requirements based on ANSI/AAMI ST91:2021. It operates at a controlled flow rate of 200 ml/min for a minimum of 2 min per flush cycle. These parameters were selected to ensure removal of residual dialysate, debris and to mitigate contamination risks, thereby improving the hygiene and safety of the tubing system between sessions. The design of the new mechanism employed in both tanks is shown in Figure 5.

The pumping of the fluid into the body and suction of the fluid out of the body is achieved via two plates mechanism. This mechanism consists of two movable plates and a fixed plate in the middle. These plates are shown in Figure 4. The fixed plate in the middle is used to support and ensure a stable movement. The upper plate is used to push the solution to the patient abdomen. It also includes heater holder covered with a metallic sheet in order to heat the solution closer to the human body temperature. The lower plate is used to produce a suction effect that will assist in taking out the solution from the patient abdomen. Detailed design of the proposed mechanism is shown in Figure 5. The movement of both plates is achieved via a stepper motor, attached to a threaded rod, and three balancing rods. These components are essential to lift and support the plates. A prototype of the proposed design was assembled, as shown in Figure 6.

### 3.2 Piping and instrumentation diagram (P&Id)

Piping and Instrumentation Diagrams (P&ID) are essential blueprints for process industries, detailing the intricate network of pipes, equipment, and controls. They serve as a universal language, facilitating clear communication among engineers globally. P&IDs play a crucial role in guiding the design, construction, and operation of industrial processes, reflecting the collective expertise and diligence of engineering teams (33, 34).

**Figure 8:**
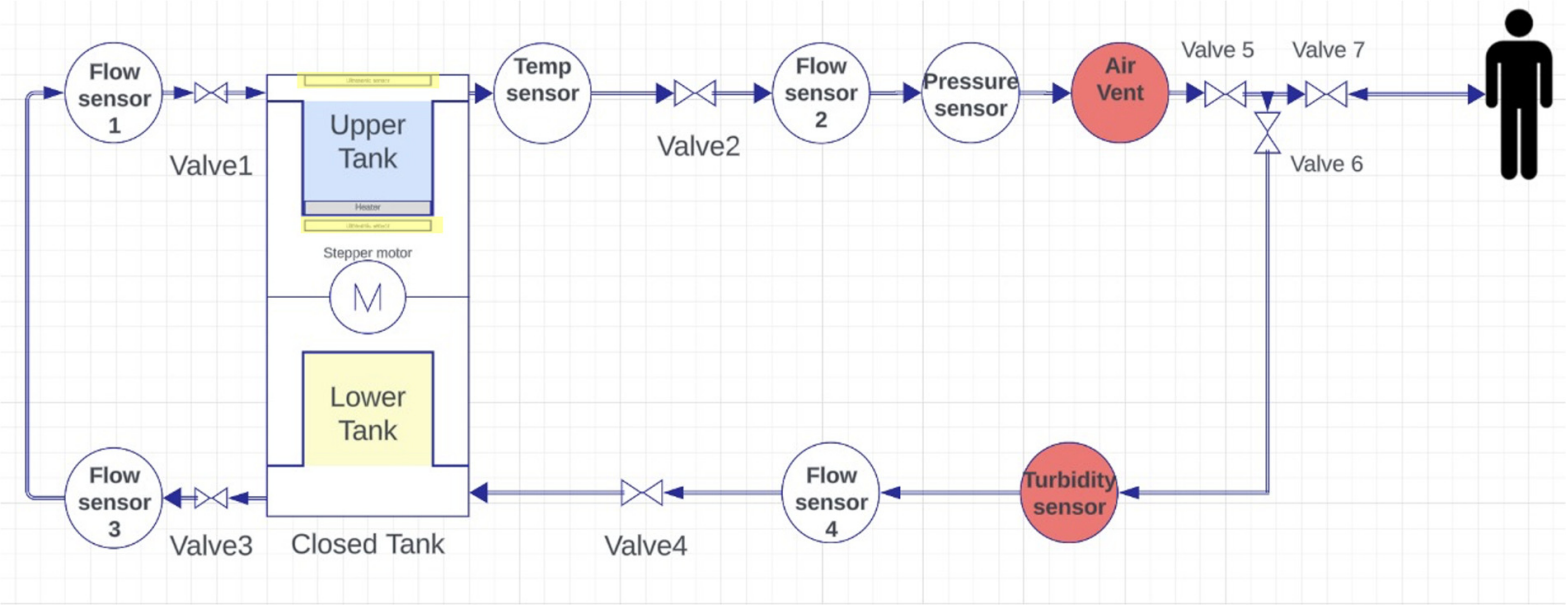
Piping and instrumentation diagram of the design.

The proposed design for the PD machine's P&ID is illustrated in Figure 8. It incorporates an upper tank with a heater to heat the solution. A temperature sensor measures the temperature, and when it reaches the desired level, valve1 opens. Flow sensor2 measures the flow rate, while a pressure sensor monitors the pressure. An air vent removes bubbles, and three valves open at specific times. Valve3 and valve1 work together in a closed loop. A turbidity sensor checks purity, and flow sensor4, followed by valve4, directs the solution to the lower tank. Finally, the solution level is monitored using two ultrasound sensors (highlighted in yellow), one placed on the cover of the upper tank and the other positioned beneath the upper tank in the middle section between the upper and lower tanks.

### 3.3 Interface design

**Figure 9:**
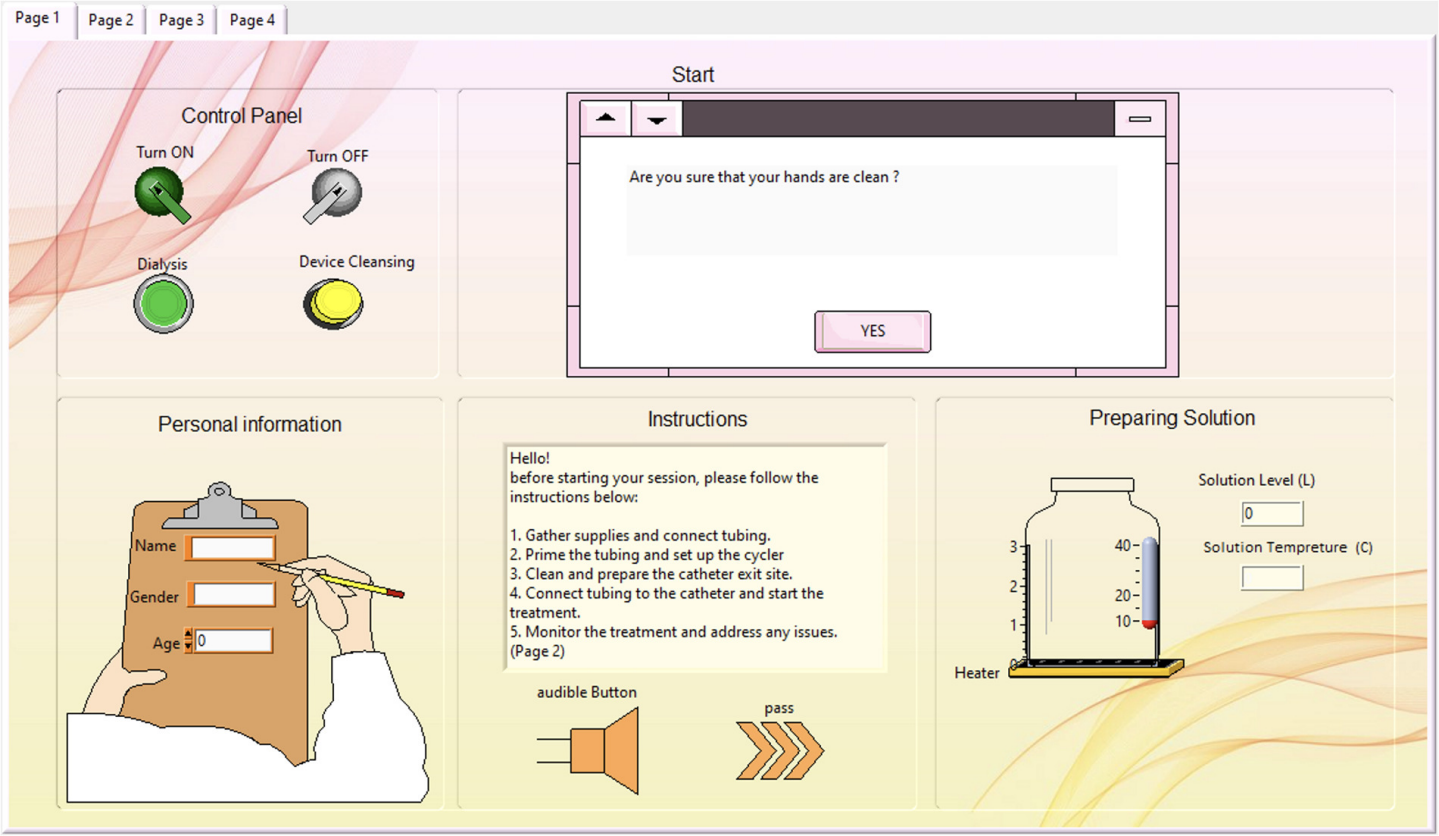
Start page of the interface window containing the control panel, personal information and safety instructions.

The simulated prototype is implemented using LabVIEW, offering a virtual user experience of peritoneal dialysis through the proposed mechanism. The front panel consists of a four-page user-friendly interface. The first page, shown in Figure 9, contains the ON/OFF buttons and options for selecting the process mode (Dialysis or Device Cleansing). Patient information must be entered, followed by safety instructions, and an audible instructions button is available if the patient desires it; otherwise, they can click “Pass.” If the user selects the dialysis mode, the device will start “Preparing the Solution,” indicating the solution level and temperature while activating the heater as needed. The last part of the start page includes three sequential questions: Are you sure your hands are clean? Is the catheter properly fixed? Are you ready to start your dialysis session? If the patient answers “yes” to all questions, the system will initiate the dialysis session and proceed to the Monitoring Panel.

**Figure 10:**
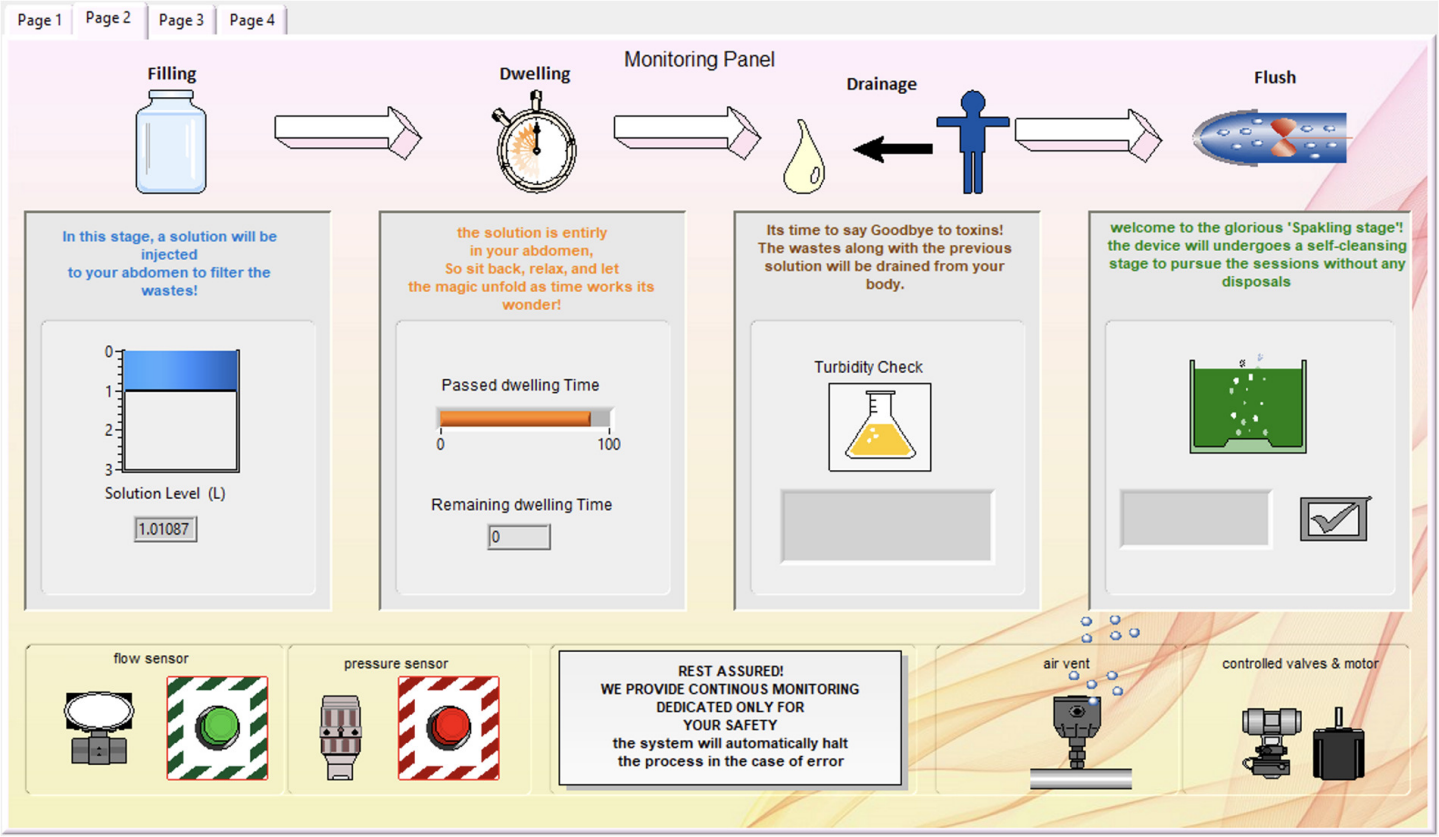
Monitoring page, where the patient can monitor the dialysis session in real-time.

In the Monitoring Panel, as illustrated in Figure 10, the patient can monitor the entire dialysis process, including the added device cleansing stage. Following that is the filling stage, where the solution level is continuously recorded to provide the patient with a view of the remaining solution.

During the dwelling phase, the duration is defined, and a timer is included. The turbidity check is integrated, with the liquid in the bottle appearing yellow to indicate high turbidity and blue to indicate low turbidity during the drainage stage.

The final part of the dialysis session is the cleansing stage, where the green tank appears sparkling until cleansing is complete. The end of the cleansing stage signifies the conclusion of the peritoneal di-alysis session. Below the stage indicators, safety measurements and device features are listed to reassure the patient, along with flow and pressure alarms. Additional safety protocols have been implemented to address potential component failures. These include redundancy checks between sensors (e.g., dual flow and pressure sensors for cross-validation), automated valve shutdowns upon abnormal readings or delayed actuation, and an emergency flush cycle triggered by a watchdog timer in case of sensor or motor malfunction. Any fault activates an error state in the FSM (state 11111), which immediately halts the system and alerts the user both visually and audibly.

**Figure 11:**
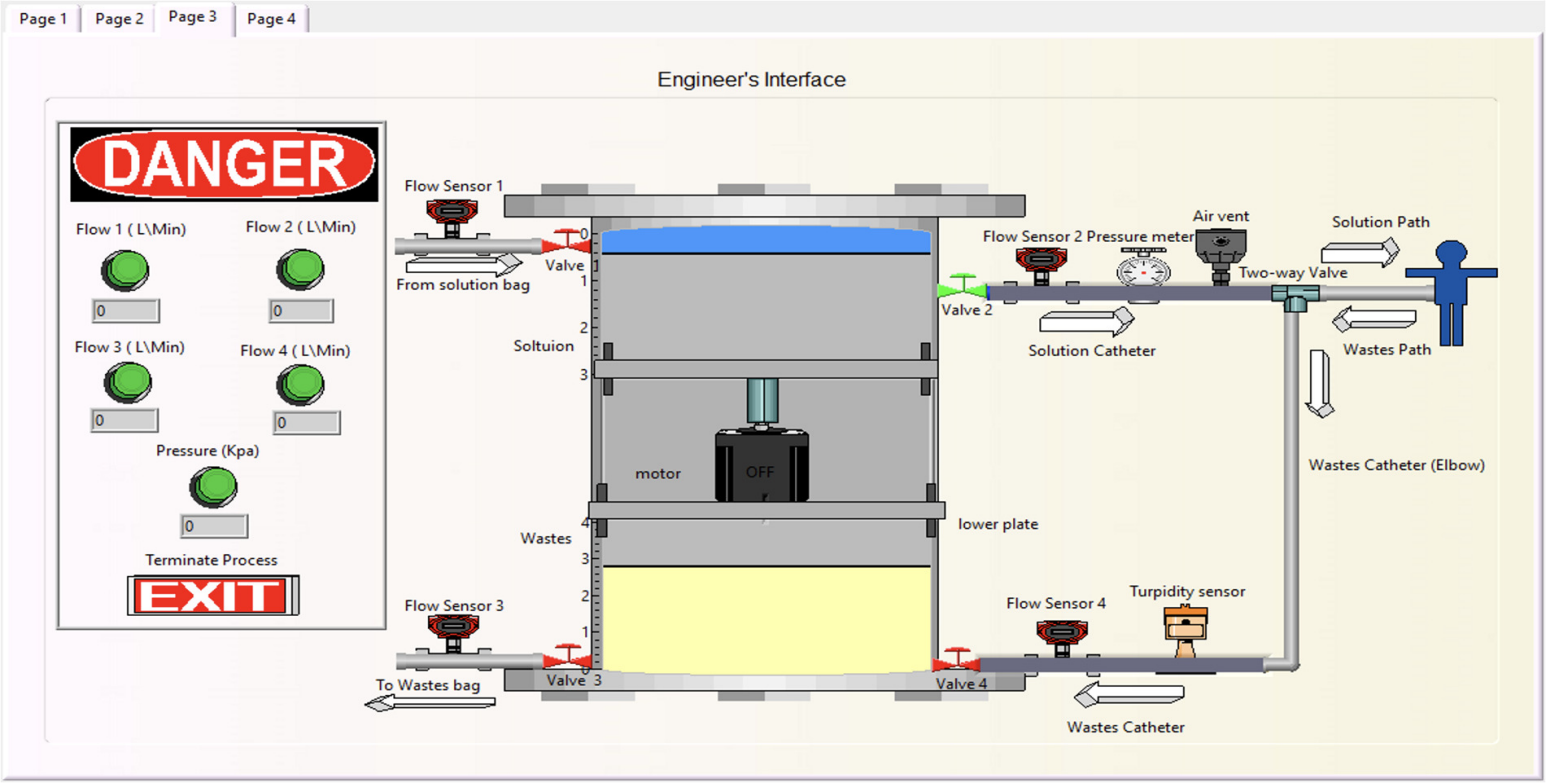
Developer's interface stage, where the engineer can modify the programming interface.

The third interface page is exclusively for the developer, as shown in Figure 11. It includes a virtual-interactive device that enables the engineer to monitor the process remotely. This page also contains detailed sensor values and alarms, along with a termination option for any errors.

**Figure 12:**
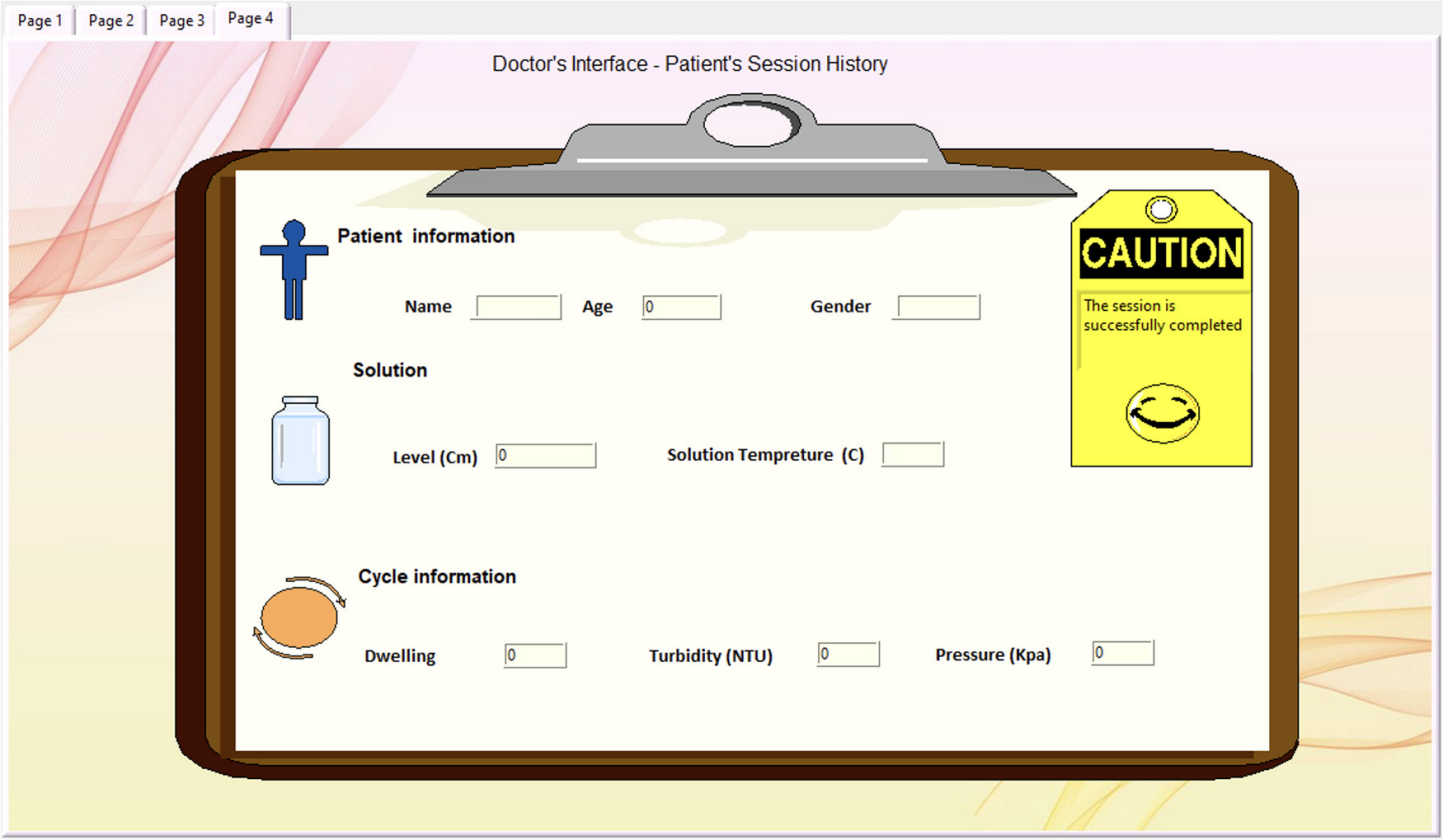
Doctor's page offering additional information exclusively for doctors.

The doctor's interface level is the last page, accessible only to doctors, illustrated in Figure 12. It is designed to provide a patient session history, assisting the doctor in reviewing necessary information with a tag indicating the success and safety of the procedure. Its primary objective is to facilitate doctors in efficiently reviewing pertinent information while providing a distinct indicator (yellow tag) to denote the success and safety of the procedure.

## 4 State machine analysis of the process

### 4.1 Finite state machine (FSM)

A Finite State Machine (FSM) consists of a limited set of states and transitions and is used to model systems that exhibit a predetermined sequence of actions (states) based on a sequence of events (transitions). FSMs can be represented using state-transition tables or state diagrams. State-transition tables contain the current state, input (transition), and next state (output). In contrast, state diagrams use nodes or squares to represent states and arrows to represent transitions between states, with labels indicating the input that triggers each transition. At any given moment, only one state is active, known as the current state. FSMs are widely used for implementing systems using processors and microprocessors and have applications in various fields (35–37). Thus, they are employed to model the process of the designed PD machine.

Finite state machine design is applied in different healthcare applications because of their ability in modeling complex systems with clear state-transition diagrams. An implementation of a hierarchical finite state (HSM) machine to control various complex processes of a hemodialysis system was presented by (38). In this work, the HSM diagram describes system behavior in a structured hierarchy of states and sub-states, each corresponding to a specific phase of the dialysis treatment such as prepare, treatment, post treatment, and alarm level state. Another example of a patent utilizes FSM was to improve the efficiency and to reduce the medical errors in infusion pumps, where is a state diagram illustrating a method of programming an infusion pump machine (39).

While the current FSM utilizes turbidity as a termination indicator, future development will include serum creatinine monitoring as a more robust physiological marker. Turbidity will act as a supporting indicator. Real-time biosensors for toxin concentration measurement are being explored to enable adaptive FSM transitions based on clearance efficiency rather than preset time-based states.

### 4.2 Input/output (I/O) description table

The I/O table, Table 2, shows that there are 16 inputs and 18 outputs representing the proposed design in Figure 3.

**Table 2:** The inputs and outputs used to represent the proposed PD design.

No.	Input	Description	Transition	Output	Description	State
1	S	Start button	T_s	SB	Stand by	S_0
2	DM	Dialysis mode button	T_dm	W.INS	Written instructions	S_1
3	FM	Flush mode button	T_fm	A.INS	Audible instructions	S_2
4	AI	Audible instructions button	T_ai	HN	Heater on	S_3
5	P	Pass button	T_p	HF	Heater off	S_4
6	T	Temperature sensor	T_t	FS	Filling start	S_5
7	SD	Start dialysis button	T_sd	FE	Filling end	S_6
8	F1	Flow sensor 1	T_f1	DWM	Downward movement	S_7
9	F2	Flow sensor 2	T_f2	DS	Draining start	S_8
10	F3	Flow sensor 3	T_f3	V4F	Valve 4 off	S_9
11	F4	Flow sensor 4	T_f4	DE	Draining end	S_10
12	L	Level sensor	T_l	LS	Loop stage	S_11
13	PR	Pressure sensor	T_pr	V31F	Valves 3 and 1 off	S_12
14	TU	Turbidity sensor	T_tu	FSH	Flush stage	S_13
15	DV	Danger value	T_dv	V456F	Valves 4, 5, and 6 off	S_14
16	F	Fault in the system	T_f	V31N	Valves 3 and 1 on + downward movement	S_15
17				V1F	Valve 1 off	S_16
18				A	Error alarm	S_17

### 4.3 State diagram

**Figure 13:**
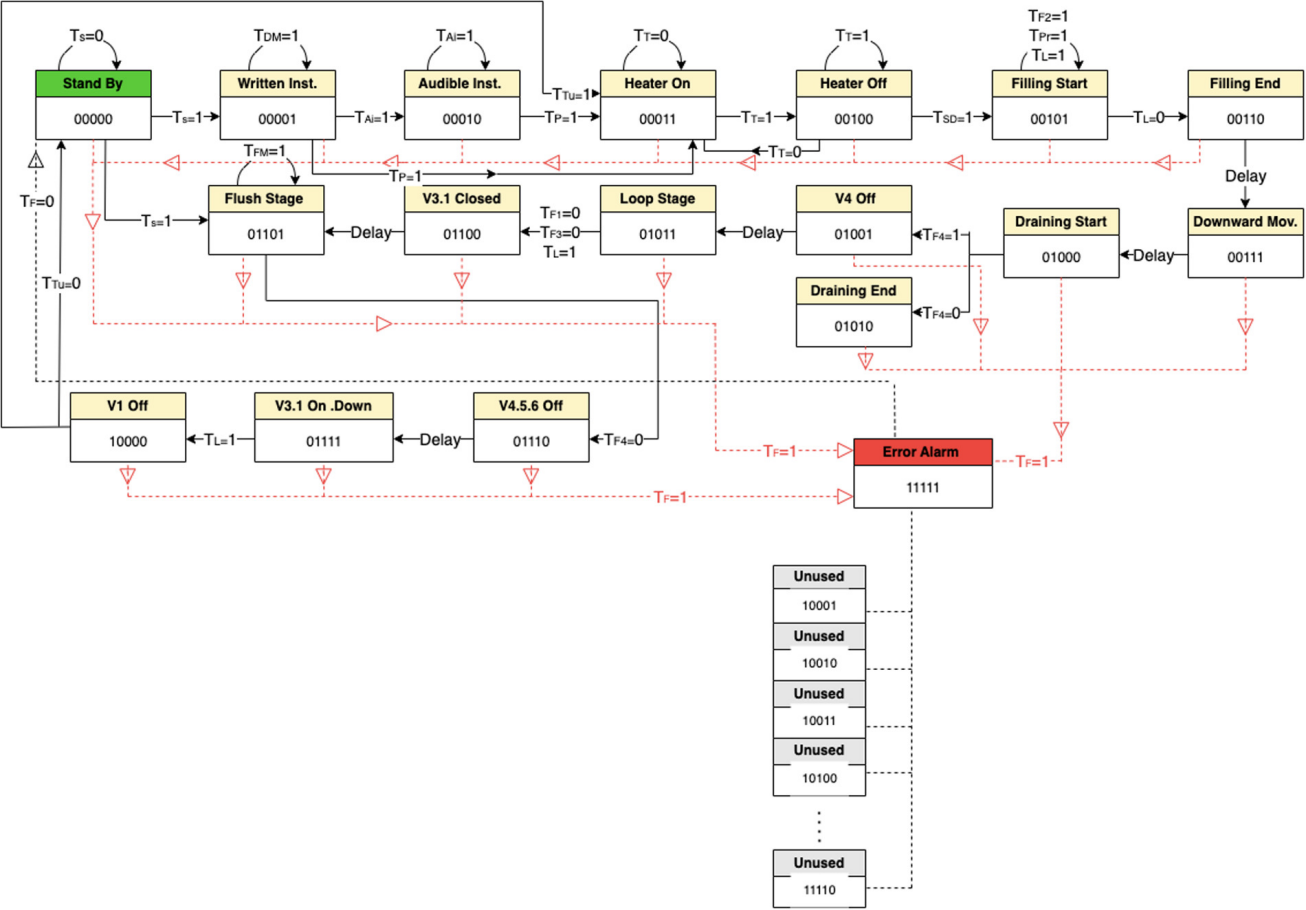
State diagram illustrating all possible states for the proposed PD process.

The state diagram presented in Figure 13 illustrates all possible states for the proposed PD process, where the standby state is assigned as 00000 and the error alarm as 11111. The states from 10001 to 11110 are not utilized.

Where:

Transition = 0 means out of range or unwanted value.

Transition = 1 means within the range or wanted value.

Number of cases $2^{5+16}=2,097,152$ cases

Where 5 (the number of flip-flops) and 16 (the total inputs).

### 4.4 State transition table and flip-flops input equations

The state transition table is used to simplify the illustration of the state, where every row has all the needed conditions for that state to be on. The following row represent the next state and so on. The unused states are annotated at the end of the Table 3.

**Table 3:** The inputs and outputs used to represent the proposed PD design.

Cases	Current state	Inputs (transitions)																Next state (output)	
	ABCDE	T_S	T_DM	T_FM	T_AI	T_P	T_T	T_SD	T_F1	T_F2	T_F3	T_F4	T_L	T_PR	T_TU	T_DV	T_F	D_A D_B D_C D_D D_E	Comment
8,192	00000	1	X	X	X	X	X	X	X	X	X	X	X	X	X	1	1	11111	S = 1, DV = 1, F = 1 go to alarm
32,768	00000	0	X	X	X	X	X	X	X	X	X	X	X	X	X	X	X	00000	S = 0 stay in stand by
2,048	00000	1	1	0	X	X	X	X	X	X	X	X	X	X	X	0	0	00001	S = 1, DM = 1, FM = 0, DV = 0, F = 0 go to w.ins
2,048	00000	1	0	1	X	X	X	X	X	X	X	X	X	X	X	0	0	01101	S = 1, DM = 0, FM = 1, DV = 0, F = 0 go to flush stage
8,192	00001	1	X	X	X	X	X	X	X	X	X	X	X	X	X	1	1	11111	F = 1 go to alarm
512	00001	1	1	0	0	0	X	X	X	X	X	X	X	X	X	0	0	00001	S = 1, F = 0, DM = 1, FM = 0, DV = 0 stay in w.ins
512	00001	1	1	0	1	0	X	X	X	X	X	X	X	X	X	0	0	00010	S = 1, DV = 0, F = 0, AI = 1 go to a.ins
1,024	00001	1	1	0	X	1	X	X	X	X	X	X	X	X	X	0	0	00011	DV = 0, F = 0, S = 1, P = 1 go to heater on
8,192	00010	1	X	X	X	X	X	X	X	X	X	X	X	X	X	1	1	11111	S = 1, DV = 1, F = 1 go to alarm
512	00010	1	1	0	1	0	X	X	X	X	X	X	X	X	X	0	0	00010	F = 0, AI = 1 stay in a.ins
1,024	00010	1	1	0	X	1	X	X	X	X	X	X	X	X	X	0	0	00011	F = 0, P = 1 go to heater on
8,192	00011	1	X	X	X	X	X	X	X	X	X	X	X	X	X	1	1	11111	F = 1 go to alarm
1,024	00011	1	1	0	X	X	0	X	X	X	X	X	X	X	X	0	0	00011	F = 0, T = 0 stay in heater on
1,024	00011	1	1	0	X	X	1	X	X	X	X	X	X	X	X	0	0	00100	F = 0, T = 1 go to heater off
8,192	00100	1	X	X	X	X	X	X	X	X	X	X	X	X	X	1	1	11111	F = 1 go to alarm
1,024	00100	1	1	0	X	X	1	X	X	X	X	X	X	X	X	0	0	00100	F = 0, T = 1 stay in heater off
1,024	00100	1	1	0	X	X	0	X	X	X	X	X	X	X	X	0	0	00011	F = 0, T = 0 go to heater on
512	00100	1	1	0	X	X	1	1	X	X	X	X	X	X	X	0	0	00101	F = 0, T = 1, SD = 1 go to filling
8,192	00101	1	X	X	X	X	X	X	X	X	X	X	X	X	X	1	1	11111	F = 1 go to alarm
128	00101	1	1	0	X	X	1	X	X	1	X	X	1	1	X	0	0	00101	F = 0, F2 = 1, L = 1, PR = 1 stay in filling
1,024	00101	1	1	0	X	X	X	X	X	X	X	X	0	X	X	0	0	00110	F = 0, L = 0 go to filling end
8,192	00110	1	X	X	X	X	X	X	X	X	X	X	X	X	X	1	1	11111	F = 1 go to alarm
2,048	00110	1	1	0	X	X	X	X	X	X	X	X	X	X	X	0	0	00111	F = 0 after filling end go to dwm with delay
8,192	00111	1	X	X	X	X	X	X	X	X	X	X	X	X	X	1	1	11111	F = 1 go to alarm
2,048	00111	1	1	0	X	X	X	X	X	X	X	X	X	X	X	0	0	01000	F = 0 after dwm go to ds with delay
8,192	01000	1	X	X	X	X	X	X	X	X	X	X	X	X	X	1	1	11111	F = 1 go to alarm
1,024	01000	1	1	0	X	X	X	X	X	X	X	1	X	X	X	0	0	01001	F = 0, F4 = 1 go to V4F
1,024	01000	1	1	0	X	X	X	X	X	X	X	0	X	X	X	0	0	01010	F = 0, F4 = 0 go to de
8,192	01001	1	X	X	X	X	X	X	X	X	X	X	X	X	X	1	1	11111	F = 1 go to alarm
2,048	01001	1	1	0	X	X	X	X	X	X	X	X	X	X	X	0	0	01011	F = 0 after V4F go to ls with delay
8,192	01010	1	X	X	X	X	X	X	X	X	X	X	X	X	X	1	1	11111	F = 1 go to alarm
8,192	01011	1	X	X	X	X	X	X	X	X	X	X	X	X	X	1	1	11111	F = 1 go to alarm
256	01011	1	1	0	X	X	X	X	0	X	0	X	1	X	X	0	0	01100	F = 0, F1 = 0, F3 = 0, L = 1 go to V31F
8,192	01100	1	X	X	X	X	X	X	X	X	X	X	X	X	X	1	1	11111	F = 1 go to alarm
2,048	01100	1	0	1	X	X	X	X	X	X	X	X	X	X	X	0	0	01101	F = 0 after V31F go to flush with delay
8,192	01101	1	X	X	X	X	X	X	X	X	X	X	X	X	X	1	1	11111	F = 1 go to alarm
2,048	01101	1	0	1	X	X	X	X	X	X	X	X	X	X	X	0	0	01101	F = 0, FM = 1 stay in flush
1,024	01101	1	0	1	X	X	X	X	X	X	X	0	X	X	X	0	0	01110	F = 0, F4 = 0 go to V456F
8,192	01110	1	X	X	X	X	X	X	X	X	X	X	X	X	X	1	1	11111	F = 1 go to alarm
8,192	01110	1	X	X	X	X	X	X	X	X	X	X	X	X	X	0	0	01111	F = 0 after V456F go to V31N with delay
8,192	01111	1	X	X	X	X	X	X	X	X	X	X	X	X	X	1	1	11111	F = 1 go to alarm
4,096	01111	1	X	X	X	X	X	X	X	X	X	X	1	X	X	0	0	10000	F = 0, L = 1 go to V1F
8,192	10000	1	X	X	X	X	X	X	X	X	X	X	X	X	X	1	1	11111	F = 1 go to alarm
1,024	10000	1	1	0	X	X	X	X	X	X	X	X	X	X	1	0	0	00011	F = 0, TU = 1 go to heater on
1,024	10000	1	1	0	X	X	X	X	X	X	X	X	X	X	0	0	0	00000	F = 0, TU = 0 go to stand by
65,536	10001	X	X	X	X	X	X	X	X	X	X	X	X	X	X	X	X	11111	Unused
65,536	11110	X	X	X	X	X	X	X	X	X	X	X	X	X	X	X	X	11111	Unused
8,192	11111	1	X	X	X	X	X	X	X	X	X	X	X	X	X	1	1	11111	F = 1 go to alarm
8,192	11111	1	X	X	X	X	X	X	X	X	X	X	X	X	X	0	0	00000	F = 0 go to stand by

The un-simplified sum of product flip-flops input equations for PD Controller are as follows:

(1) $$D_{A}=T_{DV}T_{F}+\bar{A}BCDE\;T_{S}\;T_{L\;}\bar{T_{DV}T_{F}}$$

(2) $$\;\begin{array}{r} D_{B}=T_{DV}T_{F}+\bar{ABCDE}\;T{\;}_{S}\bar{T_{DM}}T_{FM}\bar{T_{DV}T_{F}}+\bar{AB}CDE\;\;T{\;}_{S}T_{DM}\bar{T_{FM}}\;\bar{T_{DV}T_{F}}+\\ \bar{A}B\bar{CDE}\;T{\;}_{S}T_{DM}\bar{T_{FM}}\;T_{F4}\bar{T_{DV}T_{F}}+\bar{A}B\bar{CDE}\;T{\;}_{S}T_{DM}\bar{T_{FM}}\;\bar{T_{F4}}\bar{T_{DV}T_{F}}+\\ \begin{array}{c} \bar{A}B\bar{CD}E\;T{\;}_{S}T_{DM}\bar{T_{FM}}\;\bar{T_{DV}T_{F}}+\;\bar{A}B\bar{C}DE\;\;T{\;}_{S}T_{DM}\bar{T_{FM}}\bar{T_{F1}T_{F3}}T_{L}\bar{T_{DV}T_{F}}+\\ \bar{A}BC\bar{DE}\;T{\;}_{S}\bar{T_{DM}}T_{FM}\bar{T_{DV}T_{F}}+\bar{A}BC\bar{D}E\;T{\;}_{S}\bar{T_{DM}}T_{FM}\bar{T_{DV}T_{F}}+\bar{A}BC\bar{D}E\;T{\;}_{S}\bar{T_{DM}}T_{FM}\bar{T_{F4}T_{DV}T_{F}}+\\ \bar{A}BCD\bar{E}\;T_{S}\;\bar{T_{DV}T_{F}} \end{array} \end{array}$$

(3) $$\begin{array}{c} D_{C}=\\ T_{DV}T_{F}+\bar{ABCDE}\;T{\;}_{S}\bar{T_{DM}T_{FM}T_{DV}T_{F}}+\bar{ABC}DE\;T{\;}_{S}T_{DM}\bar{T_{FM}}T_{T}\bar{T_{DV}T_{F}}+\\ \bar{AB}C\bar{DE}\;T{\;}_{S}T_{DM}\bar{T_{FM}}T_{T}\bar{T_{DV}T_{F}}+\bar{AB}C\bar{DE}\;T{\;}_{S}T_{DM}\bar{T_{FM}}T_{T}T_{SD}\bar{T_{DV}T_{F}}+\\ \begin{array}{c} \;\bar{AB}C\bar{D}E\;T{\;}_{S}T_{DM}\bar{T_{FM}}T_{T}T_{F2}T_{L}T_{Pr}\bar{T_{DV}T_{F}}+\bar{AB}C\bar{D}E\;T{\;}_{S}T_{DM}\bar{T_{FM}T_{L}T_{DV}T_{F}}+\\ AB\bar{CD}E\;\;T{\;}_{S}T_{DM}\bar{T_{FM}}\;\bar{T_{DV}T_{F}}+\bar{A}B\bar{C}DE\;T{\;}_{S}T_{DM}\bar{T_{FM}T_{F1}T_{F3}}T_{L}\bar{T_{DV}T_{F}}+\\ AB\bar{CD}E\;\;T{\;}_{S}T_{DM}\bar{T_{FM}}\;\bar{T_{DV}T_{F}}+\bar{A}B\bar{C}DE\;T{\;}_{S}T_{DM}\bar{T_{FM}T_{F1}T_{F3}}T_{L}\bar{T_{DV}T_{F}}+\\ AB\bar{CD}E\;\;T{\;}_{S}T_{DM}\bar{T_{FM}}\;\bar{T_{DV}T_{F}}+\bar{A}B\bar{C}DE\;T{\;}_{S}T_{DM}\bar{T_{FM}T_{F1}T_{F3}}T_{L}\bar{T_{DV}T_{F}}+ \end{array} \end{array}$$

(4) $$\begin{array}{c} D_{D}=T_{DV}T_{F}+\bar{ABCD}E\;\;T{\;}_{S}T_{DM}\bar{T_{FM}}\;T{\;}_{Ai}\bar{\;T{\;}_{P}T_{DV}T_{F}}+\\ \bar{ABCD}E\;\;T{\;}_{S}T_{DM}\bar{T_{FM}}\;T{\;}_{P}\bar{T_{DV}T_{F}}\;\bar{ABC}D\bar{E}\;T{\;}_{S}T_{DM}\bar{T_{FM}}\;T{\;}_{Ai}\bar{\;T{\;}_{P}T_{DV}T_{F}}+\\ \begin{array}{c} \bar{ABC}D\bar{E}\;T{\;}_{S}T_{DM}\bar{T_{FM}}\;T{\;}_{P}\bar{T_{DV}T_{F}}+\bar{ABC}DE\;T{\;}_{S}T_{DM}\bar{T_{FM}}\;\bar{T_{T}T_{DV}T_{F}}\;\bar{AB}C\bar{DE}\;T{\;}_{S}T_{DM}\bar{T_{FM}}\;\bar{T_{T}T_{DV}T_{F}}+\\ \bar{ABC}D\bar{E}\;T{\;}_{S}T_{DM}\bar{T_{FM}}\;T{\;}_{P}\bar{T_{DV}T_{F}}+\bar{ABC}DE\;T{\;}_{S}T_{DM}\bar{T_{FM}}\;\bar{T_{T}T_{DV}T_{F}}\;\bar{AB}C\bar{DE}\;T{\;}_{S}T_{DM}\bar{T_{FM}}\;\bar{T_{T}T_{DV}T_{F}}+\\ \bar{ABC}D\bar{E}\;T{\;}_{S}T_{DM}\bar{T_{FM}}\;T{\;}_{P}\bar{T_{DV}T_{F}}+\bar{ABC}DE\;T{\;}_{S}T_{DM}\bar{T_{FM}}\;\bar{T_{T}T_{DV}T_{F}}\;\bar{AB}C\bar{DE}\;T{\;}_{S}T_{DM}\bar{T_{FM}}\;\bar{T_{T}T_{DV}T_{F}}+\\ A\bar{BCDE}\;T{\;}_{S}T_{DM}\bar{T_{FM}}\;T_{Tu}\bar{T_{DV}T_{F}} \end{array} \end{array}$$

(5) $$\;\begin{array}{c} D_{E}=T_{DV}T_{F}+\bar{ABCDE}\;T{\;}_{S}T_{DM}\bar{T_{FM}}\bar{T_{DV}T_{F}}+\bar{ABCDE}\;T{\;}_{S}\bar{T_{DM}}T_{FM}\bar{T_{DV}T_{F}}+\\ \bar{ABCD}E\;\;T{\;}_{S}T_{DM}\bar{T_{FM}}\bar{\;T{\;}_{Ai}}\bar{\;T{\;}_{P}T_{DV}T_{F}}+\bar{ABCD}E\;\;T{\;}_{S}T_{DM}\bar{T_{FM}}\;T{\;}_{P}\bar{T_{DV}T_{F}}+\\ \begin{array}{c} \bar{ABC}D\bar{E}\;T{\;}_{S}T_{DM}\bar{T_{FM}}\;T{\;}_{P}\bar{T_{DV}T_{F}}+\bar{ABC}DE\;T{\;}_{S}T_{DM}\bar{T_{FM}}\;\bar{T_{T}T_{DV}T_{F}}+\\ \bar{AB}C\bar{DE}\;T{\;}_{S}T_{DM}\bar{T_{FM}}\;\bar{T_{T}T_{DV}T_{F}}+\bar{AB}C\bar{DE}\;T{\;}_{S}T_{DM}\bar{T_{FM}}T_{T}T_{SD}\bar{T_{DV}T_{F}}+\\ \begin{array}{c} \bar{AB}C\bar{D}E\;T{\;}_{S}T_{DM}\bar{T_{FM}}T_{T}T_{F2}T_{L}T_{Pr}\bar{T_{DV}T_{F}}\;\bar{AB}CD\bar{E}\;T{\;}_{S}T_{DM}\bar{T_{FM}}\;\bar{T_{DV}T_{F}}+\\ \bar{A}B\bar{CDE}\;T{\;}_{S}T_{DM}\bar{T_{FM}}\;T_{F4}\bar{T_{DV}T_{F}}+\bar{A}B\bar{CD}E\;T{\;}_{S}T_{DM}\bar{T_{FM}}\;\bar{T_{DV}T_{F}}\;\bar{A}BC\bar{DE}\;T{\;}_{S}\bar{T_{DM}}T_{FM}\bar{T_{DV}T_{F}}+\\ \bar{A}BC\bar{D}E\;T{\;}_{S}\bar{T_{DM}}T_{FM}\bar{T_{DV}T_{F}}+\bar{A}BCD\bar{E}\;T_{S}\;\bar{T_{DV}T_{F}}+A\bar{BCDE}\;T{\;}_{S}T_{DM}\bar{T_{FM}}\;T_{Tu}\bar{T_{DV}T_{F}} \end{array} \end{array} \end{array}$$

## 5 Conclusions

In conclusion, there is a pressing need to improve current peritoneal dialysis devices and processes due to the increasing number of kidney failure patients and the desire to address existing limitations. Therefore, the aim of this work was to implement a state machine design for an optimized automated peritoneal dialysis process. An initial prototype was developed to test the mechanism. This successful testing without any passing solution confirmed the mechanism's effectiveness. Following the successful prototype testing, the final design was implemented. The entire electrical circuit was thoroughly tested, and the final code was verified to ensure overall accuracy. Several key innovations were incorporated into the project, including the addition of a flush system to reduce consumable materials, the design of a user-friendly interface, the inclusion of a turbidity sensor to decrease dialysis time, and a reduction in overall costs.

As a future work, the proposed design could be modified by considering different pats such as reducing the size and weight of the prototype, real time monitoring the toxin clearance efficiency by adding another sensor to check the infection of the dwelled solution not only the color, developing a mobile Applications that compatible with Android and iOS and validating the prototype by testing it for longer time with different scenarios.

## Data Availability

The raw data supporting the conclusions of this article will be made available by the authors, without undue reservation.
